# BDE-47 Disrupts Gut Microbiota and Exacerbates Prediabetic Conditions in Mice: Therapeutic Potential of Grape Exosomes and Antioxidants

**DOI:** 10.3390/toxics13080640

**Published:** 2025-07-29

**Authors:** Zaoling Liu, Fang Cao, Aerna Qiayimaerdan, Nilupaer Aisikaer, Zulipiya Zunong, Xiaodie Ma, Yale Yu

**Affiliations:** 1 State Key Laboratory of Pathogenesis, Prevention and Treatment of High Incidence Diseases in Central Asia, Xinjiang Medical University, Urumqi 830011, China; 2 School of Public Health, Xinjiang Medical University, Urumqi 830017, China

**Keywords:** 2,2′,4,4′-tetrabromodiphenyl ether (BDE-47), gut microbiota, prediabetes, environmental toxicology, metabolic dysregulation

## Abstract

**Background**: BDE-47, a pervasive environmental pollutant detected in >90% of human serum samples, is increasingly linked to metabolic disorders. This study investigates the specific impact of BDE-47 exposure on the gut microbiota in prediabetic mice and evaluates the efficacy of therapeutic interventions in mitigating these effects. **Objectives**: To determine whether BDE-47 exposure induces diabetogenic dysbiosis in prediabetic mice and to assess whether dietary interventions, such as grape exosomes and an antioxidant cocktail, can restore a healthy microbiota composition and mitigate diabetes risk. **Methods**: In this study, a prediabetic mouse model was established in 54 male SPF-grade C57BL/6J mice through a combination of high-sugar and high-fat diet feeding with streptozotocin injection. Oral glucose tolerance tests (OGTT) were conducted on day 7 and day 21 post-modeling to assess the establishment of the model. The criteria for successful model induction were defined as fasting blood glucose levels below 7.8 mmol/L and 2 h postprandial glucose levels between 7.8 and 11.1 mmol/L. Following confirmation of model success, a 3 × 3 factorial design was applied to allocate the experimental animals into groups based on two independent factors: BDE-47 exposure and exosome intervention. The BDE-47 exposure factor consisted of three dose levels—none, high-dose, and medium-dose—while the exosome intervention factor included three modalities—none, Antioxidant Nutrients Intervention, and Grape Exosomes Intervention. Fresh fecal samples were collected from mice two days prior to sacrifice. Cecal contents and segments of the small intestine were collected and transferred into 1.5 mL cryotubes. All sequences were clustered into operational taxonomic units (OTUs) based on defined similarity thresholds. To compare means across multiple groups, a two-way analysis of variance (ANOVA) was employed. The significance level was predefined at α = 0.05, and *p*-values < 0.05 were considered statistically significant. Bar charts and line graphs were generated using GraphPad Prism version 9.0 software, while statistical analyses were performed using SPSS version 20.0 software. **Results**: The results of 16S rDNA sequencing analysis of the microbiome showed that there was no difference in the α diversity of the intestinal microbiota in each group of mice (*p* > 0.05), but there was a difference in the Beta diversity (*p* < 0.05). At the gate level, the abundances of *Proteobacteria*, *Campylobacterota*, *Desulfobacterota*, and *Fusobacteriota* in the medium-dose BDE-7 group were higher than those in the model control group (*p* < 0.05). The abundance of *Patellar bacteria* was lower than that of the model control group (*p* < 0.05). The abundances of *Proteobacteria* and *Campylobacterota* in the high-dose BDE-7 group were higher than those in the model control group (*p* < 0.05). The abundance of *Planctomycetota* and *Patescibacteria* was lower than that of the model control group (*p* < 0.05), while the abundance of *Campylobacterota* in the grape exosome group was higher than that of the model control group (*p* < 0.05). The abundance of *Patescibacteria* was lower than that of the model control group (*p* < 0.05), while the abundance of Firmicutes and *Fusobacteriota* in the antioxidant nutrient group was higher than that of the model control group (*p* < 0.05). However, the abundance of *Verrucomicrobiota* and *Patescibacteria* was lower than that of the model control group (*p* < 0.05). At the genus level, the abundances of *Bacteroides* and unclassified *Lachnospiraceae* in the high-dose BDE-7 group were higher than those in the model control group (*p* < 0.05). The abundance of *Lachnospiraceae* NK4A136_group and *Lactobacillus* was lower than that of the model control group (*p* < 0.05). The abundance of Veillonella and Helicobacter in the medium-dose BDE-7 group was higher than that in the model control group (*p* < 0.05), while the abundance of Lactobacillus was lower (*p* < 0.05). The abundance of genera such as *Lentilactobacillus* and *Faecalibacterium* in the grape exosome group was higher than that in the model control group (*p* < 0.05). The abundance of *Alloprevotella* and Bacteroides was lower than that of the model control group (*p* < 0.05). In the antioxidant nutrient group, the abundance of *Lachnospiraceae* and *Hydrogenophaga* was higher than that in the model control group (*p* < 0.05). However, the abundance of *Akkermansia* and *Coriobacteriaceae* UCG-002 was significantly lower than that of the model control group (*p* < 0.05). **Conclusions**: BDE-47 induces diabetogenic dysbiosis in prediabetic mice, which is reversible by dietary interventions. These findings suggest that microbiota-targeted strategies may effectively mitigate the diabetes risk associated with environmental pollutant exposure. Future studies should further explore the mechanisms underlying these microbiota changes and the long-term health benefits of such interventions.

## 1. Introduction

According to the International Diabetes Federation (IDF) Diabetes Atlas, the global prevalence of diabetes among adults aged 20–79 years has reached 589 million, with projections indicating an increase to 853 million by 2050 (IDF Diabetes Atlas, 11th Edition) [1]. This condition not only significantly impairs the quality of life for affected individuals but also predisposes them to a range of complications, including cardiovascular diseases, ocular disorders, renal dysfunction, and neurological impairments, thereby substantially increasing the risk of disability and mortality [2]. Prediabetes, which represents a transitional stage preceding the onset of diabetes, is defined by impaired fasting glucose (IFG), impaired glucose tolerance (IGT), or a combination of both (IFG + IGT), reflecting an intermediate hyperglycemic state between normal glycemia and diabetic conditions [3]. In 2021, the age-adjusted global prevalence of IGT among adults aged 20–79 years was 9.1% (464 million), with estimates projecting an increase to 10.0% (638 million) by 2045 [4]. Additionally, in the same year, approximately 5.8% (298 million) of the global adult population within this age group exhibited IFG, a figure expected to rise to 6.5% (414 million) by 2045 [4]. Prediabetes is associated with an elevated risk of cardiovascular diseases, microvascular complications, malignancies, dementia, depression, and other health concerns. Timely and effective interventions during the prediabetic stage can significantly reduce the likelihood of progression to full-blown diabetes [5].

PBDE-47 and Metabolic Dysfunction: 2,2′,4,4′-Tetrabromodiphenyl ether (BDE-47) is one of the most commonly detected polybrominated diphenyl ethers (PBDEs) in environmental media [6]. Due to its lipophilic nature and potential for bioaccumulation, BDE-47 exerts physiological toxicity even at low concentrations. Its toxicological profile includes reproductive and developmental toxicity, thyroid disruption, neurotoxicity, and immunotoxic effects [7]. Emerging evidence indicates a positive association between exposure to BDE-47 and an increased risk of diabetes [8], highlighting the importance of investigating its impact on prediabetes and elucidating the underlying mechanisms to enhance diabetes prevention and management strategies [9].

The gut microbiota plays a crucial role in maintaining host health, and its dysregulation has been implicated in the development of various metabolic disorders [10]. In recent years, the relationship between gut microbiota and the onset and progression of diabetes has attracted significant research interest. Studies have demonstrated a strong link between gut microbiota dysbiosis and diabetes pathogenesis, revealing complex interactions involving host genetics, immune function, dietary patterns, and microbial composition [11]. Modulation of the gut microbiota has emerged as a promising therapeutic approach for diabetes management [12]. Research has shown that the gut microbial composition in individuals with type 2 diabetes differs markedly from that of healthy controls, with specific bacterial communities influencing host metabolic pathways and immune responses, thereby affecting insulin sensitivity and glucose metabolism [13].

Although the involvement of gut microbiota dysbiosis in diabetes is well established, its role in mediating the effects of environmental pollutants remains insufficiently understood. Recent studies have indicated that PBDEs can alter the gut microbiota in murine models; however, no studies have yet (1) investigated these effects specifically in the context of prediabetes or (2) evaluated microbiota-targeted interventions. We hypothesize that BDE-47 will (i) induce gut dysbiosis associated with prediabetic conditions, characterized by a reduction in mucin-degrading Akkermansia and an increase in pro-inflammatory Proteobacteria, and (ii) that these alterations can be ameliorated through the administration of anti-inflammatory grape-derived exosomes or antioxidant supplements.

## 2. Materials and Methods

### 2.1. Experimental Animals

Male C57BL/6J mice (*n* = 54, 14–16 g; Beijing Vital River) were housed in SPF conditions for 7 days at the Experimental Animal Center of Xinjiang Medical University. All experimental procedures were approved by the Animal Ethics Committee of Xinjiang Medical University and conducted in accordance with institutional guidelines.

### 2.2. Experimental Design and Methods

**Figure 1:**
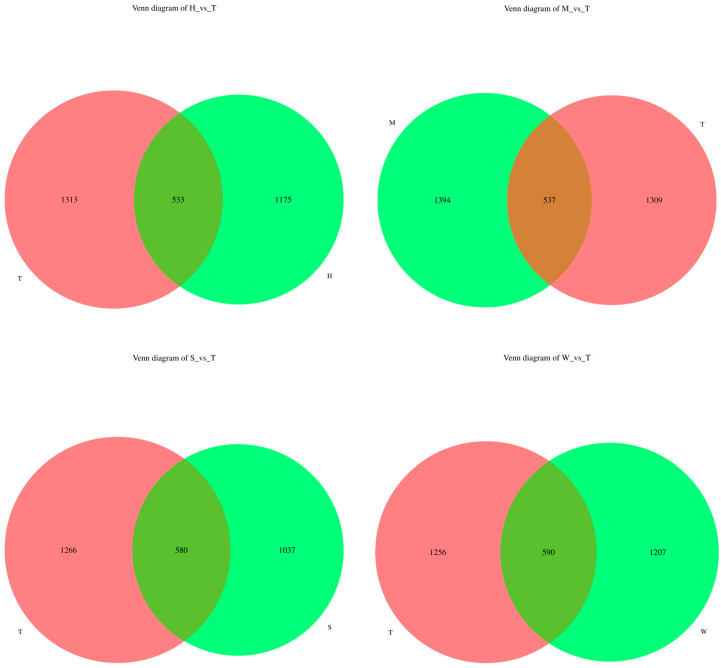
Venn diagram of ASV distribution.

In this study, a prediabetic mouse model was established in 54 male SPF-grade C57BL/6J mice. Mice were randomly assigned to nine groups (*n* = 6 per group) using a 3 × 3 factorial design, with two factors, BDE-47 exposure and exosome intervention, each at three levels. The experimental groups are summarized in Table 1. We analyzed 15 intestinal content samples, which were divided into five experimental groups: the model control group (Group T), the high-dose BDE-47 group (Group H), the medium-dose BDE-47 group (Group M), the antioxidant nutrient group (Group S), and the grape exosome group (Group W). Each group consisted of three biological replicates. The ASV distribution Venn diagram revealed that Group H and Group T shared 533 ASVs; Group M and Group T shared 537 ASVs; Group S and Group T shared 580 ASVs; and Group W and Group T shared 590 ASVs (see Figure 1).

### 2.3. Preparation of Solutions

BDE-47 Solutions: The dose of BDE-47 in the exposure groups was determined based on prior experiments conducted by our research group, as follows: high-BDE-control-dose BDE-47 exposure group (100 mg/kg) and medium-BDE-control-dose BDE-47 exposure group (50 mg/kg). Accurately weighed amounts of BDE-47 powder (50 mg and 100 mg) were completely dissolved in 10 mL of corn oil using an ultrasonic water bath. Subsequently, the mice were administered the solution via intragastric gavage at a dose of 0.1 mL/10 g body weight.

Grape Exosome Solution: Exosomes were isolated from Xinjiang Mare’s Milk grape samples using ultracentrifugation techniques and subsequently resuspended in PBS at a concentration of 0.5 mg/mL, as described in the literature [14]. The detailed procedure is outlined next. (1) Sample Pretreatment: Grape samples were weighed, peeled, washed with ultrapure water, and homogenized in PBS. The homogenate was filtered through gauze and centrifuged at 1000× *g* for 10 min to collect the supernatant. This was followed by centrifugation at 3000× *g* for 30 min to further clarify the supernatant. The sample was then concentrated using a 100 kDa ultrafiltration tube via centrifugation at 3500 rpm for 30 min. Subsequently, the supernatant was collected after centrifugation at 10,000× *g* for 30 min and filtered through a 0.22 μm microporous membrane to remove residual debris. (2) Exosome Extraction via Ultracentrifugation: The pretreated sample was subjected to ultracentrifugation at 4 °C and 100,000× *g* for 1 h. The supernatant was discarded, and the pellet was resuspended in PBS. A second ultracentrifugation step was performed at 4 °C and 100,000× *g* for 1 h to pellet the exosomes. After discarding the supernatant, the exosome pellet was resuspended in an appropriate volume of PBS. An additional low-speed centrifugation step was included to ensure removal of any remaining debris prior to exosome collection.

Antioxidant Nutrient Solution: The antioxidant nutrients selected for this study were VC, VE, and SE, which scored relatively high in the antioxidant dietary patterns previously investigated by our other research group. The dosages were determined based on the “2023 Chinese Dietary Reference Intakes for Nutrients” published by the Chinese Nutrition Society. The recommended daily intake for normal adults is 100 mg·d^−1^ for vitamin C, 14 mg·d^−1^ for vitamin E, and 60 μg·d^−1^ for selenium. These human dosages were converted to mouse dosages using a conversion factor of 9.01 for mice to humans. The final intervention dosages were set at 15.17 mg/kg for vitamin C, 2.12 mg/kg for vitamin E, and 9.1 μg/kg for selenium. The mice were administered the doses via gavage based on their body weight on the day of intervention.

### 2.4. Animal Handling

Mice were maintained under controlled conditions, with feeding, gavage, and weight measurements performed at fixed intervals throughout the study. All procedures utilized standard laboratory equipment, including a manual pipette (Eppendorf, Hamburg, Germany) and a high-speed refrigerated centrifuge (HC-3018R, Anhui Zhongke Zhongjia Scientific Instruments Co., Ltd., Hefei, Anhui Province, China).

### 2.5. Sample Collection

#### 2.5.1. Fecal Collection

Fresh fecal samples were collected from mice two days prior to euthanasia. The animals were subjected to overnight fasting but had unrestricted access to water on the day before sample collection. Feces were collected from each cage the following day and stored in 1.5 mL cryotubes at −80 °C.

#### 2.5.2. Intestinal Content Sample Collection

Cecal contents and segments of the small intestine were collected and transferred into 1.5 mL cryotubes. These samples were then immediately frozen in liquid nitrogen to preserve their structural and molecular integrity. Subsequently, the samples were stored at −80 °C in an ultra-low temperature freezer to inhibit microbial activity and prevent degradation of biomolecules during long-term storage.

### 2.6. Detection of Gut Microbiota

The bacterial 16S rDNA present in intestinal content samples was analyzed using 16S rRNA gene amplicon sequencing. Total DNA was extracted from microbial community samples using the CTAB method. The purity and integrity of the extracted DNA were assessed via agarose gel electrophoresis, while its concentration was quantified using a UV spectrophotometer. Following PCR amplification and purification of the amplicons, quality control of the PCR products was performed using an Agilent 2100 Bioanalyzer and the Kapa Biosciences Library Quantification Kit (Illumina), ensuring that library concentrations exceeded 2 nM. Qualified libraries were assigned unique indices, diluted to appropriate concentrations, and pooled according to sequencing specifications. Subsequently, the pooled libraries were converted into single-stranded DNA templates via NaOH treatment for sequencing preparation. Paired-end sequencing with a read length of 250 base pairs was carried out on the NovaSeq 6000 platform using the NovaSeq 6000 SP Reagent Kit (500 cycles).

### 2.7. Statistical Analyses

#### 2.7.1. General Data

Quantitative information within the experimental data was presented as mean values ± standard deviation. To compare means across multiple groups, a two-way analysis of variance (ANOVA) was employed. The significance level was predefined at α = 0.05, and *p*-values < 0.05 were considered statistically significant. Bar charts and line graphs were generated using GraphPad Prism version 9.0 software, while statistical analyses were performed using SPSS version 20.0 software.

#### 2.7.2. Intestinal Microbiota Sequencing Data Analysis Methods

All sequences were clustered into operational taxonomic units (OTUs) based on defined similarity thresholds. Representative sequences from OTUs with ≥97% similarity were classified using the Bayesian algorithm implemented in the RDP classifier. Microbial community composition at various taxonomic levels—including domain, kingdom, phylum, class, order, family, genus, and species—was analyzed statistically. Additionally, partial least squares discriminant analysis (PLS-DA) was conducted to evaluate inter-group sample similarities. Based on the results of OTU clustering and taxonomic classification, comprehensive analyses of microbial community structure were carried out at multiple taxonomic resolutions. These included assessments of species composition and abundance, α-diversity, β-diversity, functional abundance, and inter-group differences in functional diversity.

## 3. Results

### 3.1. Alpha Diversity Analysis and Beta Diversity Analysis

The overall diversity of gut microbiota is composed of Alpha diversity and Beta diversity.

#### 3.1.1. Alpha Diversity Analysis

Alpha diversity reveals the diversity within a given environment or ecosystem, which not only reflects the diversity and distribution balance of species, but also takes into account the depth of sequencing. Richness and evenness are usually evaluated by Chao1, Observed species, Shannon, and Simpson indices. The diversity index of each group is shown in Table 2, respectively. The results showed that there was no difference in the α diversity of intestinal flora in each group at the overall level (*p* > 0.05).

#### 3.1.2. Beta Diversity Analysis

**Figure 2:**
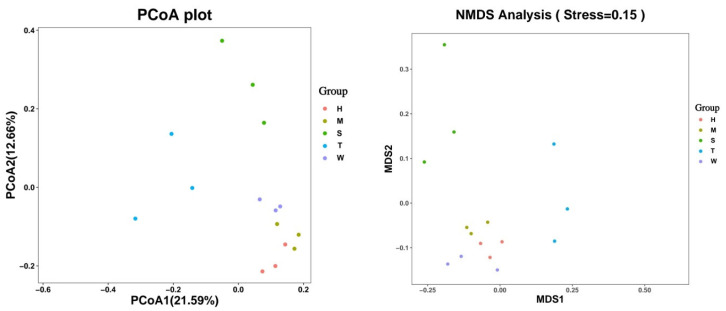
PCoA diagram and NMDS diagram.

Beta diversity describes differences in species composition between ecosystems. Together with Alpha diversity, it determines the overall biodiversity and the ecological heterogeneity of specific communities. The analysis of Beta diversity typically begins with the construction of a distance matrix that measures the distance between all sample pairs. Through Principal Component Analysis (PCA), Principal Coordinates Analysis (PCA), PCoA, Non-Metric Multidimensional Scaling (NMDS), and other methods were used to observe the differences between samples. As shown in Figure 2, points of different colors in the figure represent samples in different groups. The farther the distance is from the similarity between the reaction samples, the greater the difference is. The second principal component on the horizontal axis has a 21.59% contribution rate to the group difference, and the third principal component on the vertical axis has a 12.66% contribution rate to the group difference. The results of PCoA analysis (Bray–Curtis distance) and NMSD analysis showed that the gut microbiota of mice in the model control (T) group differed from those in the other groups (*p* < 0.05).

#### 3.1.3. Species Analysis

**Figure 3:**
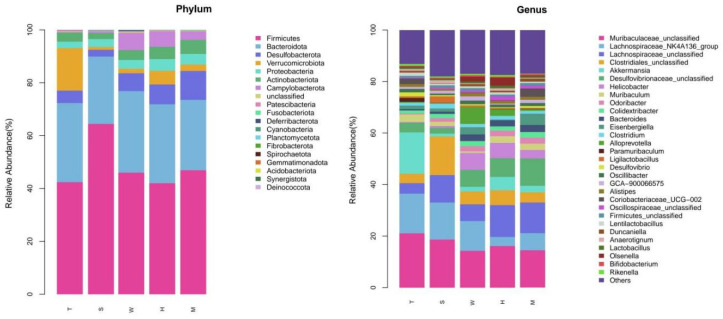
Grouped stacked bar charts of phylum level and genus level.

Using the annotation information of amplicon sequence variants (ASVs) and the abundance data of sample ASVs, species abundance tables were constructed at the phylum and genus levels. Subsequently, the species composition of different samples was analyzed at these taxonomic levels. Based on the species annotation statistics, the top 30 bacterial groups by relative abundance were selected and visualized in the form of stacked bar charts. The relative abundance profiles of the gut microbiota between the two groups at the phylum and genus levels are presented in Figure 3. All annotations are as follows: H represents the high-dose BDE-47 group, M represents the medium-dose BDE-47 group, S represents the antioxidant nutrient group, W represents the exosomal grape group, and T represents the model control group.

At the phylum level, the bacterial community compositions of each group were largely similar. The top ten phyla by relative abundance were Firmicutes, Bacteroidota, Desulfobacterota, Verrucomicrobia, Proteobacteria, Actinobacteriota, Campylobacterota, Unclassified, Patescibacteria, and Fusobacteriota. Comparing the relative abundances of the top five phyla, the abundance of Desulfobacterota was higher in both the high-dose BDE-47 group (H group) and the medium-dose BDE-47 group (M group). With increasing BDE-47 dosage, the relative abundances of Desulfobacterota and Campylobacterota increased, whereas the relative abundance of Verrucomicrobia decreased. In the exosomal grape group (W group), the relative abundances of Firmicutes and Desulfobacterota were slightly elevated. Conversely, the model control group (T group) exhibited higher relative abundances of Bacteroidota and Proteobacteria. Additionally, the relative abundance of Verrucomicrobia was higher in the model control group (T group) compared to the exosomal grape group (W group). Notably, the antioxidant nutrient group (S group) demonstrated the highest relative abundance of Firmicutes.

At the genus level, the top ten genera by relative abundance were Muribaculaceae Unclassified (Muribaculaceae_unclassified), Lachnospiraceae NK4A136_group (Lachnospiraceae_NK4A136_group), Lachnospiraceae Unclassified (Lachnospiraceae_Unclassified), Clostridiales Unclassified (Clostridiales_Unclassified), Akkermansia (Akkermansia), Desulfovibrionaceae Unclassified (Desulfovibrionaceae_Unclassified), Helicobacter (Helicobacter), Muribaculum (Muribaculum), Odoribacter (Odoribacter), and Colidextribacter (Colidextribacter). Comparing the top five genera by relative abundance, the relative abundance of Lachnospiraceae NK4A136_group decreased in the high-dose BDE-47 group (H group). In the grape exosome group (W group), the relative abundances of Lachnospiraceae_Unclassified (unclassified rumen bacteria family) and Clostridiales_Unclassified (unclassified Clostridium order) were slightly higher. Conversely, the relative abundances of Muribaculaceae_Unclassified (unclassified murine bacteria family), Lachnospiraceae_NK4A136_group (ruminant bacteria family of NK4A136 group), and Akkermansia (Akkermansia genus) were slightly lower in the grape exosome group (W group). In the antioxidant nutrient group (S group), the relative abundance of Clostridiales_Unclassified (unclassified Clostridium order) increased.

These results suggest that variations in BDE-47 dosage result in distinct alterations in gut microbiota composition, with differential impacts on microbial structure observed across the treatment groups. These insights lay a solid foundation for further investigations into the complex interactions between gut microbiota and environmental factors.

#### 3.1.4. Analysis of Differences in Gut Microbiota Structure

**Figure 4:**
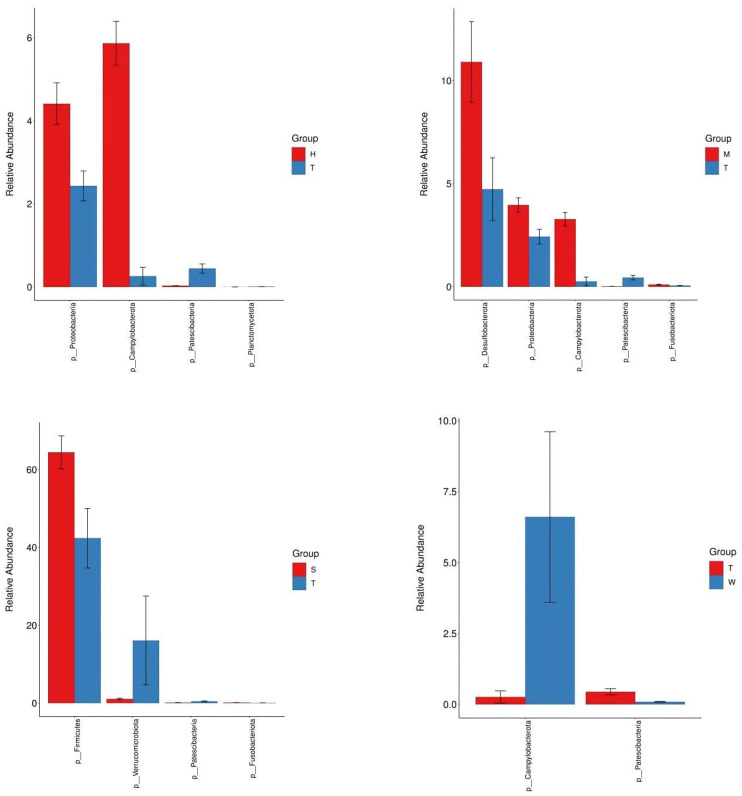
Phylum-level difference analysis bar plot.

The results of the 16S rDNA sequencing differential analysis revealed that, at the phylum level, the abundance of Desulfobacterota, Proteobacteria, Campylobacterota, and Fusobacteriota in the medium-dose BDE-47 group (M group) was significantly higher than that in the model control group (*p* < 0.05), whereas the abundance of Patescibacteria was significantly lower (*p* < 0.05). In the high-dose BDE-47 group (H group), the abundance of Proteobacteria and Campylobacterota was significantly higher than that in the model control group (*p* < 0.05), while the abundance of Planctomycetota and Patescibacteria was significantly lower (*p* < 0.05). In the grape exosome group (W group), the abundance of Campylobacterota was significantly higher than that in the model control group (*p* < 0.05), whereas the abundance of Patescibacteria was significantly lower (*p* < 0.05). In the antioxidant nutrient group (S group), the abundance of Firmicutes and Fusobacteriota was significantly higher than that in the model control group (*p* < 0.05), while the abundance of Verrucomicrobiota and Patescibacteria was significantly lower (*p* < 0.05). For detailed information, see Figure 4.

At the genus level, the high-dose BDE-47 group (Group H) showed increased abundances of Lachnospiraceae_unclassified, Bacteroides, Murimonas, Klebsiella, and Gardnerella (*p <* 0.05), while Desulfovibrionaceae_unclassified, Lachnospiraceae_NK4A136_group, Lactobacillus, and others were less abundant (*p* < 0.05). The medium-dose BDE-47 group (Group M) exhibited higher abundances of Veillonella and Helicobacter (*p* < 0.05), with reduced abundances of Desulfovibrio, Lactobacillus, and others (*p* < 0.05).

**Figure 5:**
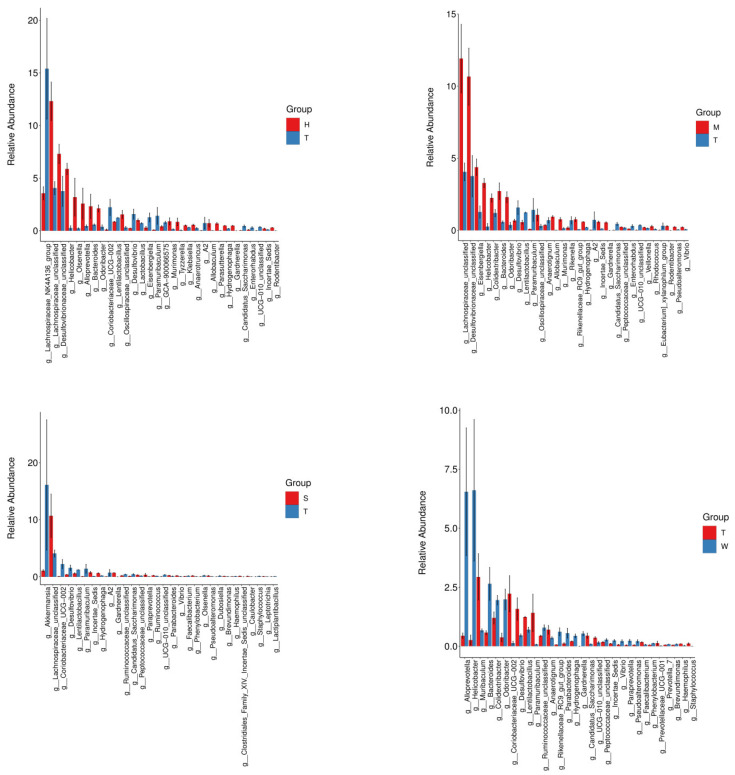
Genus-level difference analysis bar plot.

In the Glucose Exosome group (Group W), the abundances of Muribaculum, Coriobacteriaceae_UCG-002, Desulfovibrio, and Lentilactobacillus were elevated (*p* < 0.05). Additionally, genera such as Bacteroides (Fecalibacterium), Prevotella_UCG-001 (Prevotellaceae), Haemophilus, and Staphylococcus were more abundant than in the control group (*p* < 0.05), while Alloprevotella, Helicobacter, Bacteroides, Colidextribacter, Odoribacter, Ruminococcaceae_unclassified, Rikenellaceae_RC9_gut_group, Parabacteroides, Hydrogenophaga, Gardnerella, Peptococcaceae_unclassified, Incertae_Sedis, Vibrio, Paraprevotella, Pseudoalteromonas, and Phenylbacterium were less abundant (*p* < 0.05). In the antioxidant nutrient group (Group S), the abundances of Lachnospiraceae and Hydrogenophaga were significantly higher (*p* < 0.05), whereas Akkermansia and Coriobacteriaceae_UCG-002 were less abundant (*p* < 0.05). A detailed elucidation of these results is provided in Figure 5.

### 3.2. Prediction of Gut Microbiota Function

**Figure 6:**
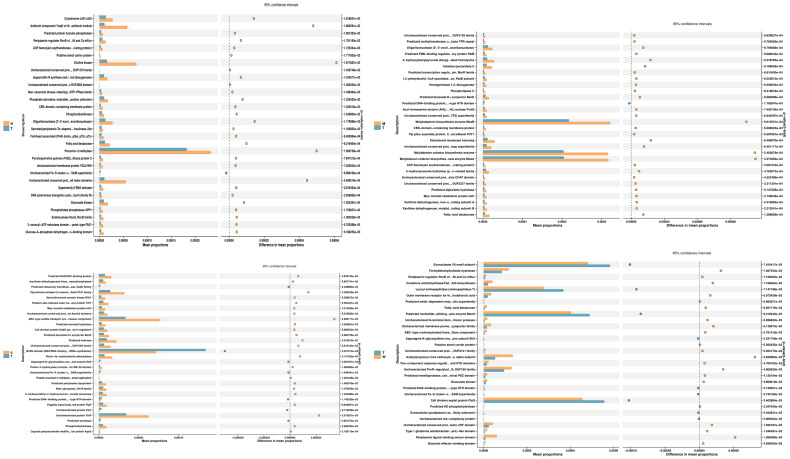
Bar graph of the COG database annotated metabolic prediction results.

PICRUSt (Phylogenetic Investigation of Communities by Reconstruction of Unobserved States) establishes a “mapping” between flora and function, which is the best choice for predicting flora function. Functional prediction analysis using PICRUSt2 software can provide gene family-based annotation results covering multiple databases, including Clusters of Orthologous Groups (COGs) and KEGG Orthology (KO). Such analyses help to understand the underlying functional properties of microbial communities. H is the BDE-47 high-dose group, M is the BDE-47 medium-dose group, S is the antioxidant nutrient group, W is the grape exosome group, and T is the model control group. Annotation of the results from the COG database shows that, compared with the T group, the antitoxin component YwqK of the antitoxin module, Choline kinase, and Precorrin−2, in group H, the metabolites of methylase (preporphyringeno-2 methylase) were statistically different; In group M, Molybdopterin biosynthesis enzyme MoaB (molybdenum cofactor biosynthesis enzyme MoaB), molybdenum cofactor biosynthesis enzyme (molybdenum cofactor biosynthesis enzyme), and molybdenum The metabolites of the cofactor biosynthesis enzyme MoaA were statistically different. B3/B4 domain (DNA/RNA-binding tRNA-synthetase) (B3/B4 domain (DNA/RNA-binding tRNA-synthetase)), ABC-type sulfate transport system permease in S group component (permease component of ABC-type sulfate transport system), and uncharacterized protein YhfF (uncharacterized protein YhfF) metabolites were statistically different, the Exonuclease VII small subunit (Exonuclease VII) in group W Small subunit), Leucyl aminopeptidase (aminopeptidase T), and predicted nucleotide-utilizing enzyme MoeA (predicted nucleotide utilization enzyme MoeA) metabolites were statistically different, and the results are shown in Figure 6.

## 4. Discussion

In the present investigation, a prediabetic murine model was meticulously established to systematically explore the impact of 2,2′,4,4′-tetrabromodiphenyl ether (BDE-47) on the gut microbiota. Rigorous experimental analyses demonstrated that exposure to BDE-47 induced a statistically significant and profound alteration in the composition of the murine gut microbiota. At the phylum taxonomic level, administration of BDE-47 led to a notable augmentation in the relative abundances of Desulfobacterota and Proteobacteria, whereas a concomitant decrease was observed in the abundance of Verrucomicrobia. Intriguingly, these observations are consistent with previous reports documenting an elevated relative abundance of these phyla in the intestinal microbiota of diabetic patients. Such congruence strongly implies that these microbiota compositional changes may be mechanistically linked to the pathogenesis of diabetes, as dysbiosis of the gut microbiota has been well-documented to disrupt metabolic homeostasis and adversely impact metabolic health [15].

At the genus taxonomic level, exposure to BDE-47 induced significant alterations in the relative abundances of specific bacterial genera. In the high-dose BDE-47 experimental group, a marked increase was observed in the abundance of the genus Faecalibaculum [16], which has been associated with gut microbiota dysbiosis linked to metabolic disorders. These taxonomic changes may be closely related to the etiopathogenesis of diabetes. Notably, while specific genera such as Bacteroides, Murimonas, Klebsiella, and Gardnerella were not explicitly mentioned in the provided literature, other studies have demonstrated that an elevated Bacteroidetes/Firmicutes ratio, potentially influenced by BDE-47 exposure through mechanisms like enhancing the abundance of Bacteroidetes, is a characteristic feature of diabetes in mice [17,18] and may exacerbate metabolic abnormalities. Furthermore, the substantial decrease in the abundances of unclassified Desulfovibrionaceae, the Lachnospiraceae NK4A136 group [16,19], and Lactobacillus [20] highlights the potential of BDE-47 exposure to increase the risk of diabetes. Such disruptions in these specific microbial populations could disturb the delicate ecological balance of the gut microbiome, leading to metabolic dysregulation and facilitating the development of diabetic phenotypes.

The Lachnospiraceae family, which belongs to the Clostridiales order, plays a critical role in the biosynthesis of short-chain fatty acids (SCFAs), key metabolites essential for maintaining gut homeostasis and regulating host metabolic processes [21]. SCFAs, primarily generated via the fermentation of dietary fibers by commensal gut microbiota, function as vital energy sources for colonic epithelial cells [22], modulate gut barrier integrity [23], and exhibit anti-inflammatory properties [24]. Furthermore, they are crucial for regulating glucose and lipid metabolism [22,25], enhancing insulin sensitivity [26], and maintaining energy homeostasis in the host.

Lactobacillus, a well-recognized genus of probiotic bacteria, is central to maintaining gut health. These bacteria contribute significantly to the modulation of gut microbiota composition, enhancement of gut barrier integrity, regulation of metabolic pathways, and modulation of the immune system. By producing antimicrobial substances, competing for nutrients and adhesion sites, and modulating the host immune response, Lactobacillus strains help maintain a balanced gut microbiota and prevent the overgrowth of potentially pathogenic bacteria.

The observed decrease in the abundance of Lactobacillus in the context of BDE-47 exposure may have far-reaching consequences. A reduction in Lactobacillus populations can compromise gut barrier function, allowing increased translocation of lipopolysaccharides and other microbial products into the bloodstream, which in turn triggers a systemic inflammatory response. This chronic low-grade inflammation is closely associated with the development of insulin resistance, a key pathophysiological feature of type 2 diabetes. Moreover, alterations in Lactobacillus abundance may disrupt the normal metabolic functions of the gut microbiota, leading to dysregulation of SCFA production and other metabolic pathways, further contributing to the development of metabolic disorders such as diabetes [27].

The discernible augmentation in the abundance of Lachnospiraceae within the antioxidant nutrient group strongly implies that these bacterial genera may confer benefits by enhancing gut barrier integrity, attenuating inflammatory responses, and bolstering host metabolic well-being [28]. Mechanistically, Lachnospiraceae are proficient producers of short-chain fatty acids (SCFAs), such as butyrate, propionate, and acetate. Butyrate, in particular, serves as a primary energy source for colonic epithelial cells, promotes tight junction protein expression, and thereby fortifies the gut barrier, preventing the translocation of harmful pathogens and endotoxins. Additionally, SCFAs exert anti-inflammatory effects by modulating immune cell function and inhibiting the production of pro-inflammatory cytokines.

Conversely, the decline in the abundance of Desulfovibrionaceae may have significant implications for host metabolic regulation. This family of bacteria is known to be involved in the metabolism of sulfur-containing compounds and has been linked to the regulation of gut-derived hormones. Specifically, a reduction in Desulfovibrionaceae may disrupt the production of glucagon-like peptide-1 (GLP-1), a key incretin hormone that plays a crucial role in glucose homeostasis. Research has demonstrated that certain species within the Desulfovibrio genus can inhibit GLP-1 production through the release of hydrogen sulfide. This inhibition impairs insulin secretion, glucose uptake, and satiety regulation, ultimately contributing to the development of metabolic disorders, including obesity [29].

When comparing the medium-dose BDE-47 group with the model control group, distinct shifts in the gut microbiota composition were evident. Several genera, including *Veillonella* and *Helicobacter*, exhibited increased abundances, while *Desulfovibrio* and *Lactobacillus* showed significant decreases. The upsurge in *Veillonella* has been implicated in diabetic pathophysiology. *Veillonella* is capable of metabolizing lactate produced by other bacteria into acetate, which may influence energy metabolism and contribute to insulin resistance. Moreover, the increase in *Helicobacter* has been associated with diabetes and its associated complications. *Helicobacter* infection can trigger chronic inflammation, disrupt gut microbiota balance, and interfere with metabolic pathways, thereby exacerbating the risk of developing diabetes and its associated comorbidities [30,31].

The results of the study demonstrated that interventions with graphene-based engineered living nanomaterials (GELNs) and antioxidant nutrients led to a notable increase in the relative abundance of Firmicutes within the gut microbiome. As a major phylum of bacteria, Firmicutes play an indispensable role in the host’s energy metabolism and the production of short-chain fatty acids (SCFAs) [32]. These SCFAs, including acetate, propionate, and butyrate, are not only important energy sources for host cells but also key regulators of various physiological processes, such as modulating gut barrier function, influencing immune responses, and participating in metabolic regulation.

Inter-group microbiota differential analysis further revealed a significant upregulation of the abundance of *Faecalibacterium* in the GELNs intervention group. *Faecalibacterium* is renowned for its ability to produce high levels of butyrate, a particularly beneficial SCFA. Through the production of butyrate, *Faecalibacterium* may exert positive effects on gut barrier function by promoting the integrity of intestinal epithelial tight junctions, thereby preventing the leakage of harmful substances from the gut lumen into the bloodstream. Additionally, butyrate can modulate the immune system, reducing chronic inflammation and enhancing immune cell function [33].

Collectively, these findings strongly suggest that environmental pollutants have the potential to disrupt the composition of the gut microbiota. Such alterations in the gut microbiome can then trigger a cascade of effects, ultimately impacting host health [34]. The dysbiosis caused by environmental pollutants may lead to imbalances in metabolic processes, compromised gut barrier function, and dysregulated immune responses, all of which are associated with the development of various diseases.

The present study further revealed that interventions involving grape exosomes and antioxidant nutrients were capable of attenuating the alterations in the gut microbiota induced by 2,2′,4,4′-tetrabromodiphenyl ether (BDE-47). Grape exosomes, nanosized extracellular vesicles secreted by grape cells, are postulated to modulate the composition of the gut microbiota through their inherent anti-inflammatory and antioxidant properties. By scavenging reactive oxygen species (ROS) and suppressing the activation of pro-inflammatory signaling pathways, grape exosomes may counteract the detrimental effects of BDE-47 on the gut microbial ecosystem, thereby alleviating the toxic impacts of this environmental pollutant.

The efficacy of antioxidant nutrient interventions provides additional support for this conclusion. These nutrients, which include vitamins (such as vitamin C and E), polyphenols, and other bioactive compounds, are known to enhance the antioxidant defense system of the host. By increasing the levels of antioxidant enzymes (such as superoxide dismutase, catalase, and glutathione peroxidase) and reducing oxidative stress, antioxidant nutrients can mitigate the inflammation and oxidative damage caused by BDE-47 exposure. This, in turn, may contribute to the restoration of the gut microbiota to a more balanced state, thereby protecting the host from the adverse health consequences associated with BDE-47-induced gut dysbiosis.

## 5. Conclusions

In the present study, a prediabetic murine model was rigorously established to systematically investigate the effects of 2,2′,4,4′-tetrabromodiphenyl ether (BDE-47) on the gut microbiota and its potential role in diabetes progression. The results clearly demonstrate that BDE-47 exposure induced significant alterations in both the structure and functional profile of the gut microbiota in mice, particularly at the phylum and genus taxonomic levels. Specifically, the relative abundances of Desulfobacterota and Proteobacteria were markedly increased, while that of Verrucomicrobia was reduced in the BDE-47-treated groups. These microbial shifts have been well-documented to be closely associated with the pathogenesis of diabetes. Furthermore, BDE-47 exposure led to notable changes in specific bacterial genera, including significant decreases in Lachnospiraceae and Lactobacillus.

Alpha diversity analysis revealed no statistically significant differences in species richness or evenness among the experimental groups, suggesting that short-term BDE-47 exposure and interventions did not substantially affect these diversity indices. However, beta diversity analysis using Principal Coordinate Analysis (PCoA) and Non-Metric Multidimensional Scaling (NMDS) demonstrated a clear separation between the BDE-47 treatment group and the control group, indicating a significant alteration in microbial community structure (*p* < 0.05). At the phylum level, the abundance of Desulfobacterota and Proteobacteria increased with escalating BDE-47 doses, whereas Verrucomicrobia exhibited a dose-dependent decrease. At the genus level, significant changes were observed, including elevated levels of Lachnospiraceae_unclassified, Bacteroides, Murimonas, Klebsiella, and Gardnerella in the high-dose BDE-47 group, alongside reductions in Desulfovibrionaceae_unclassified, Lachnospiraceae_NK4A136_group, and Lactobacillus.

This investigation further revealed that interventions employing grape-derived exosomes and antioxidant nutrients effectively ameliorated BDE-47-induced gut microbiota dysbiosis. The grape exosome intervention was associated with increased abundances of beneficial genera such as Muribaculum and Coriobacteriaceae_UCG-002, while the antioxidant nutrient group showed enhanced levels of Lachnospiraceae and Hydrogenophaga. Functional profiling via PICRUSt2 analysis identified substantial modifications in microbial metabolic pathways, including upregulation of stress response mechanisms and disruption of cofactor-mediated metabolic processes following BDE-47 exposure. These findings suggest that dietary or nutritional interventions may serve as promising strategies for mitigating the adverse effects of environmental pollutants on the gut microbiota.

In conclusion, this study highlights the significant impact of BDE-47 on the gut microbiota composition and function in prediabetic mice, offering novel insights into how environmental contaminants may contribute to diabetes risk through microbiota-mediated mechanisms. The findings underscore the importance of minimizing exposure to environmental pollutants and maintaining a balanced gut microbiota as preventive measures against prediabetes. These insights hold considerable value for informing future environmental health policies and advancing effective strategies for diabetes prevention.

## Figures and Tables

**Table 1:** Experimental design showing PBDE-47 exposure levels * and intervention groups in mice.

Group Number	Group Labels	BDE-47 Exposure	Exosome Intervention
1	Control	None	None
2	H-BDE-Control	High-Dose	None
3	M-BDE-Control	Medium-Dose	None
4	Antioxidant-Control	None	Antioxidant Nutrients Intervention
5	H-BDE-Antioxidant	High-Dose	Antioxidant Nutrients Intervention
6	M-BDE-Antioxidant	Medium-Dose	Antioxidant Nutrients Intervention
7	Grape-Control	None	Grape Exosomes Intervention
8	H-BDE-Grape	High-Dose	Grape Exosomes Intervention
9	M-BDE-Grape	Medium-Dose	Grape Exosomes Intervention

* The units of PBDE-47 dosing: µg/kg/day.

**Table 2:** Alpha diversity index comparison.

Alpha Diversity Index	Group T	Group W	Group S	Group H	Group M	*p*-Value
observed_otus	924.00 ± 180.47	874.67 ± 140.91	783.33 ± 199.83	876.67 ± 49.32	963.67 ± 88.93	0.630
shannon	6.92 ± 1.12	7.44 ± 0.09	6.85 ± 1.07	7.30 ± 0.29	7.59 ± 0.06	0.655
simPson	0.94 ± 0.08	0.98 ± 0.01	0.95 ± 0.04	0.98 ± 0.01	0.98 ± 0.00	0.609
Chao1 Index	925.73 ± 182.31	875.23 ± 141.36	783.92 ± 199.42	876.88 ± 49.60	964.03 ± 89.07	0.632

## Data Availability

The datasets generated and analyzed during this study are available under restricted access to protect ongoing research collaborations. Researchers may request access to the raw 16S rRNA sequencing data (FASTQ files), processed OTU tables, and associated metadata by contacting the corresponding author at [zaoling.liu@gmail.com]. Requests will be evaluated based on scientific merit and compliance with institutional data transfer agreements.
